# Susceptibility of Women to Cardiovascular Disease and the Prevention Potential of Mind–Body Intervention by Changes in Neural Circuits and Cardiovascular Physiology

**DOI:** 10.3390/biom11050708

**Published:** 2021-05-10

**Authors:** Hyun-Jeong Yang, Eugene Koh, Yunjeong Kang

**Affiliations:** 1 Korea Institute of Brain Science, Seoul 06022, Korea; chiw55@naver.com; 2 Department of Integrative Health Care, University of Brain Education, Cheonan 31228, Korea; 3 Temasek Life Sciences Laboratories, Singapore 117604, Singapore; eugene@tll.org.sg

**Keywords:** women, cardiovascular disease, mind–body intervention

## Abstract

Women have been reported to be more vulnerable to the development, prognosis and mortality of cardiovascular diseases, yet the understanding of the underlying mechanisms and strategies to overcome them are still relatively undeveloped. Studies show that women’s brains are more sensitive to factors affecting mental health such as depression and stress than men’s brains. In women, poor mental health increases the risk of cardiovascular disease, and conversely, cardiovascular disease increases the incidence of mental illness such as depression. In connection with mental health and cardiovascular health, the presence of gender differences in brain activation, cortisol secretion, autonomic nervous system, vascular health and inflammatory response has been observed. This connection suggests that strategies to manage women’s mental health can contribute to preventing cardiovascular disease. Mind–body interventions, such as meditation, yoga and qigong are forms of exercise that strive to actively manage both mind and body. They can provide beneficial effects on stress reduction and mental health. They are also seen as structurally and functionally changing the brain, as well as affecting cortisol secretion, blood pressure, heart rate variability, immune reactions and reducing menopausal symptoms, thus positively affecting women’s cardiovascular health. In this review, we investigate the link between mental health, brain activation, HPA axis, autonomic nervous system, blood pressure and immune system associated with cardiovascular health in women and discuss the effects of mind–body intervention in modulating these factors.

## 1. Susceptibility of Women to Cardiovascular Disease

### 1.1. Gender Differences in Mortality from Cardiovascular Disease

Accumulated research has shown more negative clinical outcomes from cardiovascular disease (CVD) in women compared to men. Epidemiological studies show that the CVD mortality rate in women exceeds that found in men [1,2,3]. In patients with acute coronary syndrome (ACS) who undergo primary percutaneous coronary intervention, the mortality rate is higher in women compared with men of similar age [4,5]. Moreover, after myocardial infarction, younger women have a higher mortality rate than men of the same age or older women during hospitalization [6]. Coronary microvascular dysfunction (CMVD) which is associated with hyperactivation of the sympathetic nervous system is more frequently observed in women than in men, which increases the event-free mortality rate in women, but not in men [7,8,9].

### 1.2. Stress and Cardiovascular Disease

A large-scale prospective follow-up data analysis in adults has shown that stress is associated with CVD [10,11,12,13]. Adulthood stress plays an important role as a disease stimulator for individuals who already have a high burden of arteriosclerosis plaque. It correlates with increased risk of negative clinical outcomes for those suffering from preexisting conventional cardiovascular/cerebrovascular diseases [12]. According to a meta-analysis of stress as a predictor of CVD and mortality [12], while the link between adulthood stress, cardiovascular and cerebrovascular events is moderate in the general public, strong links are found in high-risk groups experiencing partner bereavement, anger and emotional instability [14,15,16,17], and stressors are strongly associated with recurrent cardiovascular events and mortality in patients with CVD [18,19,20,21,22]. Thus, adulthood stress plays an important role in the progression and outcome of CVD [12].

### 1.3. Relationship between Mental Health and Cardiovascular Health and Its Gender Differences 

Persistent stress reactions are related to the onset and maintenance of mental health problems such as anxiety and depression [23,24,25]. Chronic stress and exposure to adverse conditions during early development have been found to be highly correlated with the onset of depression [26]. Depression is twice as common in women as in men [27,28], the frequency of which starts to diverge from mid-puberty and is maintained into later life [29]. Depression is associated with the development of CVD [30], and women are more prone to develop depression-related CVD [31]. There is growing evidence that patients with depressive disorder (DD) are more likely to develop acute myocardial infarction, heart failure or stroke [32,33]. In a 16-year follow-up study, women with depression were more likely to develop myocardial infarction or stroke [34]. Anxiety, one of the risk factors of CVD, is more likely to occur in women than in men throughout their lives, with a male to female ratio of 1:1.7 [35], and is related with major cardiac events in patients with coronary heart disease (CHD) [36].

In takotsubo cardiomyopathy which results in temporary left ventricular dysfunction as a result of severe psychological stress, the hypoconnectivity of brain structures related to autonomic nervous system control is observed in patients [37]. The decrease in estrogen levels in postmenopausal women increases the susceptibility of takotsubo cardiomyopathy [38], and women over 55 years of age are almost five times more likely to develop takotsubo cardiomyopathy than women under 55 years of age [39]. After mental stress, women exhibited more negative and fewer positive feelings than men and higher platelet aggregation and developed more cases of myocardial ischemia caused by mental stress [40]. In the Women’s Health Initiative which involved 93,676 postmenopausal women for four years, depression was found to be an independent risk factor for CVD death and all-cause mortality after adjusting for other existing risk factors such as age, race, diabetes, hypertension, smoking, high cholesterol, etc. among women with no history of CVD [41].

Conversely, CVD affects mental health, especially in women. Compared to men, women experience twice the prevalence of major depressive disorder (MDD) after stroke [42]. After myocardial infarction, young women are twice as likely to develop mental stress-induced myocardial ischemia as men of the same age [43]. 

### 1.4. Effects of Menopause on Women’s Cardiovascular Health

Unlike men, women experience dramatic changes in the secretion of sex hormones during menopause, thus experiencing physiological and psychological changes. Middle-aged women experience a decrease in estradiol levels as they pass menopause, and the pattern of decline varies slightly from woman to woman and also changes dynamically [44]. Estradiol has been shown to exert protective effects against cardiovascular disease [45], but ongoing studies have reported contradictory results in the effect of estradiol patterns on arteriosclerosis in women during menopausal transition [46]. The level of the follicle-stimulating hormone (FSH) starts to increase six years before the final menstrual period [47,48], but the pattern of hormone dynamics depends on various factors such as race/ethnicity and premenopausal body mass index (BMI) [44]. While not all studies show connections between the FSH and subclinical CVDs, significant associations have been consistently found. In middle-aged women, higher level of FSH correlated with thicker carotid intima media thickness (cIMT) [49], lower brachial artery flow-mediated dilatation (FMD) [50] and a greater number of aortic plaques [51].

In addition, a number of risk factors for CVD increase with respect to the onset of menopause in women. In the middle age around menopause, the levels of total cholesterol, triglycerides, apolipoprotein B and low-density lipoprotein cholesterol increase significantly [52,53,54], which correlates with the presence of the carotid plaque after menopause [55]. Menopause transition is also associated with adverse vascular remodeling which accompanies changes in carotid intima media thickness and carotid adventitial diameter [56,57,58]. Blood pressure of women is generally lower than that of men before menopause, but prevalence of hypertension, a major risk factor for cardiovascular disease, is higher than that of men after menopause [59].

Type 2 diabetes mellitus is an established risk factor for CVD [60]. In a population-based cross-sectional study which investigated the association between menopause and type 2 diabetes among 8191 middle-aged women (median age, 56), the postmenopausal status was found to be the most important risk factor for type 2 diabetes in women [61]. 

Meta-analysis of menopausal initiation and CVD risk has consistently shown that early menopause initiation increases the CVD risk [62,63,64,65]. In a 2015 meta-analysis study which involved 297,496 women with early menopause onset in 31 studies, early menopause onset was associated with a higher risk of overall coronary heart disease (CHD), fatal CHD, CVD mortality and all-cause mortality [63]. In another 2019 meta-analysis which included 301,438 women in 15 studies, premature menopause (<40 years) or early menopause (40–44 years) were related with a higher CVD risk compared to women with normal menopause (50–51 years). On the other hand, women who reached menopause at age >51 years had a significantly reduced CVD risk [65].

## 2. Link between the Brain and Cardiovascular Health

**Figure 1:**
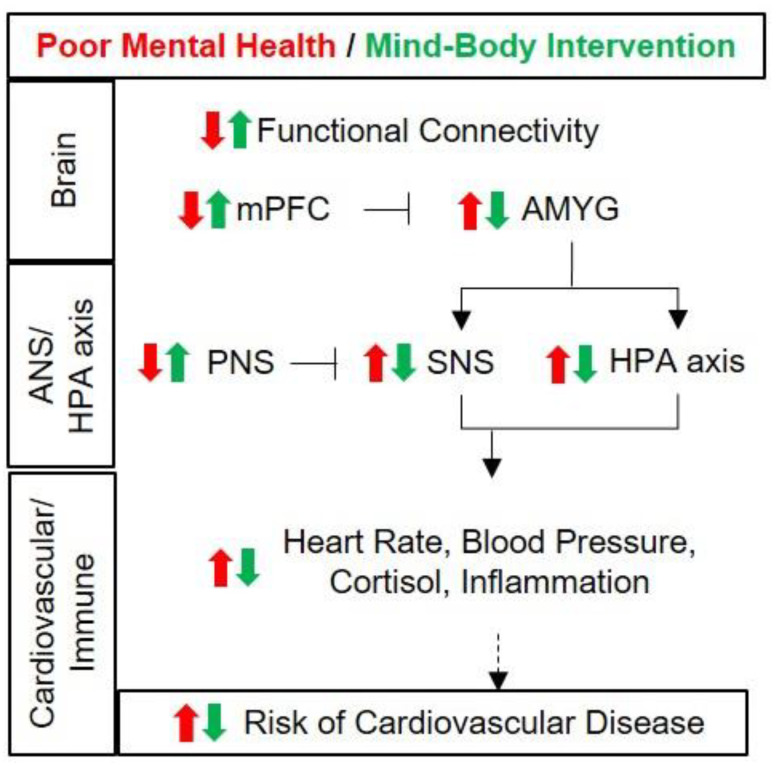
Potential mechanism of reduction in cardiovascular disease risks by mind–body intervention in women. Unlike in men, significant associations between amygdala activity and physiological responses have been reported specifically in women [72,73,74,75]. Poor mental health (red), such as chronic persistence of depression and stress, reduces functional connectivity between the prefrontal cortex and the amygdala, decreases activity of the medial prefrontal cortex and increases activity of the amygdala, which is controlled by the prefrontal cortex. Subsequently, the sympathetic nervous system is activated to increase the heart rate and blood pressure, while the HPA axis is activated to increase cortisol secretion, and the chronic activation of these states induces inflammation. The above states represent increased risks for cardiovascular disease. On the other hand, the application of mind–body intervention (green) helps improve mental health by reducing perceived stress and depression. The functional connectivity between the prefrontal cortex and the amygdala increases, and the activity of the medial frontal cortex also increases, thereby reducing the activity of the amygdala. Mind–body intervention activates the parasympathetic nervous system, thereby reducing sympathetic nerve activity, as well as reducing HPA axial activity as stress decreases. As a result, heart rate, blood pressure and cortisol secretion are reduced and inflammatory conditions are reduced. These states mean a reduction in cardiovascular disease risk. AMYG, amygdala; mPFC, medial prefrontal cortex; SNS, sympathetic nervous system; HPA, hypothalamus–pituitary–adrenal cortex.

When the brain is stressed, changes occur in the stress response neural circuit [66]. The sympathetic nervous system and hypothalamus–pituitary–adrenal cortex (HPA) axis are activated, which increases the levels of epinephrine, norepinephrine and cortisol, inducing elevation of heart rate and blood pressure [67]. Chronic exposure to stress reduces activity of the medial prefrontal cortex, resulting in overactivation of the amygdala. This induces continuous activation of the noradrenaline-producing locus coeruleus as well as chronic activation of the sympathetic nervous system [68]. Subsequently, this aggravates the inflammatory process which is called sterile inflammation (inflammation induced in the absence of pathogens) [69] and promotes endothelial dysfunction and arteriosclerosis [70] (Figure 1). This neural circuit–inflammatory–vascular link differs between women and men and disproportionately contributes to cardiovascular risk [71].

### 2.1. Sexual Dimorphism in the Connection between the Brain and Cardiovascular Health

Several studies suggest that the amygdala is an important central nervous system structure in gender-specific CVD events. In healthy adults, resting state functional connectivity (rsFC) of the amygdala differ in women and in men. Compared to men, women exhibit higher left amygdala rsFC with other structures such as the hippocampus, inferior frontal gyrus, left middle temporal gyrus and postcentral gyrus [76]. In a four-year follow-up study of 300 participants (median age, 55 years), the higher the resting metabolic activity in the amygdala, the higher the risk of developing cardiovascular disease was [77]. Amygdala hyperactivity is connected to preclinical atherosclerosis [78] and major depression [79]. In addition, overactivation of the amygdala is directly related to myocardial injury in women but not in men [8,74,75]. Cardiac autonomic dysregulation in women with major depressive disorders and negative emotional stimulation is associated with hypoconnectivity between the hippocampus, amygdala, right orbitofrontal cortex and hypothalamus [72]. In the same subgroup, hyperactivity of the right amygdala and hypothalamus was observed.

### 2.2. Gender Differences in Stress-Induced HPA Axis Activation

Within minutes of exposure to stressors, the HPA axis is activated, which releases the corticotropin-releasing hormone (CRH) from the hypothalamus into the anterior pituitary gland [80], and the CRH stimulates the pituitary gland to release the adrenocorticotropic hormone (ACTH) into the bloodstream. The ACTH causes the adrenal cortex to secrete the glucocorticoid cortisol [81]. Under stress, when glucocorticoid levels increase, inflammatory cytokines increase [82], and these are regulated by the negative feedback of the glucocorticoid [83]. However, chronic activation of the stress response can inhibit the negative feedback mechanism [83] and contribute to the initiation of disease, mental illness and poor wellbeing [23,84,85]. 

Since gender differences are found in stress-related diseases [86], it is estimated that there will be gender differences in the regulation of the HPA axis by stress. Indeed, differences in the response of the HPA axis to stress are observed in humans [87]. The prevalence of stress-related diseases (e.g., major depressive disorders, affective disorders) developed after puberty is higher in women [86,88,89], while physiological stress responses before puberty are comparable between the genders [90]. However, from adolescence into adulthood, studies on sex differences in HPA axis responses are less consistent [86]. For example, there are studies in which adult women have higher HPA responses to acute stressors compared to men, while others exhibit no significance in the responses [86,87,91,92,93]. The inconsistency is presumably because of factors including age, overall health and menstrual cycle stage [86,91,93]. When investigating cortisol responses in women at various stages of the menstrual cycle and men, gender differences were found in saliva cortisol responses to psychosocial stress when women were in the low-estrogen state of the follicular phase but not in the luteal phase [94]. Thus, estrogen and progesterone are suggested to play a role in controlling stress reactivity across the menstrual cycle in women. There is also a gender difference in the secretion of cortisol, a product of the HPA axis. According to a survey of 204 healthy adults (60 men), women have higher morning cortisol secretion (when adjusted to sex hormones) than men [95]. In addition, in women, cortisol is negatively associated with the rsFC of the amygdala with brain regions related with emotion, reward, memory processing and action execution, compared to the positive association in men [76].

### 2.3. Gender Differences in Stress-Induced Changes in the Autonomous Nervous System 

The amygdala has a strong projection effect to the brain stem (e.g., locus coeruleus, pons) and produces sympathetic neural responses to threat and stress in the brain stem [96]. The sympathetic nervous system has a direct cardiostimulatory effect and a pressor effect. The chronic imbalance of the autonomous nervous system in the form of increased sympathetic tone and decreased parasympathetic tone is a strong risk factor for morbidity of the cardiovascular system and mortality [97]. In the general population, the long-term effects of dysfunction on both the autonomic nervous system and the HPA axis contribute to changes that accelerate arteriosclerosis [66]. Patients with depression exhibit excessive stimulation of the sympathetic nervous system and decreased parasympathetic tone [98].

Short sleep and fragmented rest are often seen in depressed women, which prevents control of the autonomic nervous system and increases heart rate and blood pressure in association with cortisol hypersecretion [99]. Several studies have suggested that DD is associated with greater sympathetic nervous system activation and decreased regulation of the parasympathetic nervous system of the heart rate and blood pressure [32,33]. This phenomenon may be most relevant to women, in whom the imbalance in autonomous functions is shown to be worse than that of men [99]. In women with CMVD, takotsubo cardiomyopathy [100,101] and ACS [102], an increase in sympathetic tone and negative cardiovascular outcomes were observed [103]. Heart failure and myocardial infarction have worse prognosis in women, which is associated with upregulated cardiac sympathetic nervous activity [104,105].

### 2.4. Relation of Stress Activation of the HPA Axis and Cardiovascular Health in Women

The stress-induced autonomic nervous system and HPA axis activity affect hemostatic factors, increasing platelet activity, fibrinogen levels, viscosity and coagulation factors [12]. In the long run, it promotes arteriosclerosis [66] and triggers the occurrence of cardiovascular/cerebrovascular events, which negatively affect the cardiovascular system [106]. Serotonin plays a pivotal role in platelet aggregation, and women have a higher platelet aggregation response to the serotonin and epinephrine circulation level than men [40]. After mental stress, women experienced more cases of myocardial ischemia and exhibited more negative feelings and fewer positive feelings and higher collagen-stimulated platelet aggregation [40]. Depression is reported to be associated with higher endothelial damage in adolescent females [107].

The basic structure and mechanism for the cardiovascular system is different between genders. The epicardial coronary arteries are smaller in women than in men regardless of adjustment of the body mass index, left ventricular size and age [108]. As the myocardial blood flow in women is higher than that in men, the overall coronary flow reserve in women and men is comparable [109,110]. However, this is thought to give a higher endothelial shear stress in the coronary arteries of women [111]. Moreover, cholesterol production and metabolism, which are important determinants of vascular health, seem to be affected by gender differences as well [112,113]. Middle-aged women who reported vascular motor symptoms (i.e., hot flushes or night sweats) were at an increased risk of coronary heart disease within the subsequent 10–15 years [114,115]. A cross-sectional study which investigated 1906 postmenopausal women indicated that vasomotor symptoms were associated with metabolic syndrome, which is related with cardiovascular risk [116].

### 2.5. Gender Differences in Inflammation and Their Relation with the Cardiovascular System

Inflammation plays an important role in the development and progression of arteriosclerosis [117]. Increased stress-induced activity in the amygdala was related to increased secretion of the inflammatory cytokine interleukin (IL)-6 [118]. The sympathetic nervous system affects metabolism (promoting insulin resistance and lipolysis) and the immune system (increasing inflammation) [119,120,121,122,123]. Specifically, the sympathetic nervous system induces systemic stress-induced sterile inflammation (elevation of inflammatory proteins in the absence of pathogens), which is passed from the circulatory system to tissues, and increases inflammatory danger/damage-associated molecular patterns (DAMPs) and reduces anti–inflammatory miRNA [69].

Chronic exposure to stressors increases cortisol secretion and inflammatory reactions simultaneously. As a result, it contributes to stress response exhaustion, chronic low-grade inflammation and antigen-specific immunosuppression [69]. Cortisol was originally thought to be anti-inflammatory, but it was reported that its in vitro administration induced increased cytokine production and activation of NF-kB in isolated macrophages [124]. When cortisol is pre-dosed to healthy individuals, production of inflammatory cytokine IL–6 was increased in the continual endotoxin challenge response [125]. In mental illnesses such as clinical anxiety and depression, increase in inflammatory cytokines due to stress was observed [82,83,126,127].

Sexual dimorphism is observed in both innate and acquired immunity [128]. Inflammation is the immune system’s response to stimulation, and gender differences in inflammation are found across the course of life [129]. In the case of chronic inflammation, probably due to collateral tissue damage, women have worse prognosis and higher mortality than men. In detail, higher mortality is observed in women suffering from cystic fibrosis [130], chronic obstructive pulmonary disease [131], as well as worse prognosis in girls suffering from chronic asthma, cystic fibrosis and sickle cell anemia [132].

Inflammation can at least partially affect the development of coronary artery disease (CAD) in depressed women. In depressed patients, increased levels of acute-phase proteins (e.g., C-reactive protein (CRP), α-1-acid glycoprotein, α 1-antichymotrypsin, haptoglobin), cell adhesion molecules and circulating cytokines are commonly observed [133]. Unlike in men, increases in the body mass index (BMI) and other CVD risk factors in women are closely related to higher inflammation states (=higher CRP level) [134,135,136,137]. In female patients with impaired heart function, the upregulation of amygdala metabolism is positively associated with increased inflammation. However, this connection is not found in male patients [75]. Furthermore, the IL-6 reaction to mental stress is higher in young women with CAD compared to men of the same age [138]. This suggests that the interaction between psychological stress, myocardial damage and inflammation has characteristics of sexual dimorphism.

## 3. Changes in the Brain and Physiological Responses by Mind–Body Intervention

Multiple lifestyle factors such as diet, physical activity, tobacco, treatment plan adherence, stress and coping have complex effects on chronic disease such as cardiovascular disease [139,140,141,142]. Mind–body interventions help reduce stress [143,144] and also produce beneficial effects on unhealthy behaviors such as overeating and smoking that can be induced by stress [145,146]. In addition, mind–body interventions themselves have also been shown to exert positive effects on physical health [147,148]. This suggests that mind–body interventions can have a positive effect on the increase in healthy lifestyle factors, resulting in at least a partial contribution to the prevention of cardiovascular disease. Indeed, an increase in the number of healthy lifestyle factors are related with a decrease in the risk of cardiovascular disease [142]. Since previous reviews and recent meta-analysis of the effects of lifestyle factors on cardiovascular disease are well-reported [139,140,141,142], in this section, we limit the discussion to the scope of the effect of mind–body interventions on cardiovascular disease. In addition, although the effects of mind–body intervention for women have been reported only in a limited number of studies, its potential for CVD prevention is critical. Therefore, here, we investigate the effects of mind–body interventions in women and focus on the aspects of the brain, autonomic nervous system, HPA axis and cardiovascular and immune systems as discussed above (Figure 1, Table 1).

### 3.1. Effects of Mind–Body Intervention on Cardiovascular Disease

A 2017 American Heart Association scientific statement on meditation and cardiovascular risk suggested that meditation could help reduce cardiovascular risk and therefore be considered as an addition to guidance-oriented cardiovascular risk reduction interventions. Meditation can increase physical and mental relaxation, resulting in improved outcomes after major cardiovascular events [149]. Meditation effects are assumed to be mainly mediated by the hypothalamus–pituitary–adrenal axis, hypothalamus–pituitary–thyroid axis, renin–angiotensin–aldosterone system and energy homeostasis, and changes in the endocrine function following meditation are estimated to correspond to improvements in mental health [150]. In studies using the 2012 and 2017 National Health Interview Survey data with a total of 61,267 participants, the relationship between meditation and cardiovascular risk in patients who reported health issues such as hypercholesterolemia, systemic hypertension, diabetes, stroke and CAD was investigated. In this study, it was found that meditation was independently associated with lower prevalence rates of hypercholesterolemia, diabetes, stroke or CAD compared to those who did not meditate after adjusting for age, gender, BMI, race, marital status, smoking, sleep time and depression [151].

**Table 1:** Changes in psychological state, heart rate, blood pressure, vasomotor state, cortisol and cytokine secretion by mind–body interventions focusing on women.

Scope	Sub-Scope	Reference	Study Type	Population (*n*, % Female, Age)	Intervention	Control	Considered Confounders	Outcome
Brain (psychological)	Depression	Gong et al. [152]	Meta-analysis (six RCTs)	Pregnant women (*n* = 375, 100% female, age range, 20–40)	Yoga	Usual care or any other physical or mental care	N/A	Compared to the comparison groups, the level of depression was significantly reduced in yoga groups.
Brain (psychological)	PTSD	Van der Kolk et al. [153]	RCT	Women with chronic, treatment-resistant PTSD (*n* = 64, 100% female, mean age, 43)	Yoga for ten weeks	Health education for ten weeks	Age, race, education, marital status, income, etc.	Both groups exhibited significant decreases on the PTSD scale, with a larger reduction in the yoga group compared to the control group.
Brain (psychological)	Depression, stress, anxiety	Haller et al. [154]	Meta-analysis (ten RCTs)	Women with breast cancer (*n* = 1709, 100% female)	MBSR, MBCT	Usual care, active comparator (supportive expressive therapy, nutritional education program)	N/A	Compared to usual care, there were significant postintervention effects of MBSR/MBCT for health-related QOL, fatigue, sleep, stress, anxiety, and depression. Compared to other active interventions, significant effects were found for anxiety and depression.
Brain (psychological)	Affect (female vs. male)	Kang et al. [155]	RCT	Sixth-grade students (*n* = 114, 46% female, mean age, 12)	School-based mindfulness training for six weeks	Active control for six weeks	Age, % female, psychological state	Female meditators exhibited greater increases in positive affect compared to females in the control group, whereas male meditators and control males showed equivalent gains. Increases in self-reported self-compassion were associated with improvements in affect among females but not males.
Brain (psychological)	Affect (female vs. male)	Rojiani et al. [156]	A longitudinal study	University students (*n* = 77, 47% female, mean age, 21)	Meditation for 12 weeks	N/A	Age, affect, mindfulness, self-compassion, placebo effect-like confounders driven by self-selection	Women exhibited greater decreases in negative affect and greater increases in mindfulness and self-compassion compared to men.
Brain (psychological)	Anxiety, withdrawal symptoms (female vs. male)	Chen et al. [157]	A controlled longitudinal study	Volunteers in the rehabilitation unit of a residential addiction treatment facility (*n* = 207, 27% female, mean age, 34)	Qigong meditation (relaxation, breathing, guided imagery, inward attention, mindfulness) for two weeks	Stress management and relaxation training for two weeks	Race, % female, employment, education, social perception (religion, general feeling about life, etc.), withdrawal symptoms, etc.	Female meditation participants reported a significantly higher reduction in anxiety and withdrawal symptoms than did any other group.
Brain (structure)	Brain structure (female vs. male)	Luders et al. [158]	A cross-sectional study	Long-term meditators (mean practice time, 20.2 years) vs. meditation-naïve individuals; mean age, 47 years; 50% female; *n* = 60	N/A	N/A	Sex, handedness, age	Meditation effects differed between men and women in magnitude, laterality and location on the hippocampus surface.
Brain (psychological)	Depression, anxiety	Wong et al. [159]	RCT	Postmenopausal women with mild to moderate symptoms (*n* = 197, 100% female, mean age, 52)	MBSR for eight weeks	Menopause education for eight weeks	Age, education, occupation, marital status, religion, family size, income, menopause state	MBSR showed a greater reduction of psychological symptoms of depression and anxiety than active controls but did not reduce other somatic, urogenital and vasomotor symptoms.
Cardiovascular	Vasomotor symptoms	Chattha et al. [160]	RCT	Women with menopausal symptoms (*n* = 120, 100% female, mean age, 48)	Yoga (postures, breathing, meditation) for eight weeks	Exercise (walking, stretching, rest) for eight weeks	Age, occupation, BMI, diet, menopause state	Hot flushes, night sweats and sleep disturbance were significantly reduced in the yoga group compared to the control group.
Cardiovascular	Vasomotor symptoms	Carmody et al. [161]	RCT	Late perimenopausal and early postmenopausal women experiencing moderate or severe hot flushes (including night sweats) (*n* = 110, 100% female, mean age, 53)	MBSR for three months	Waitlist	Age, race, education, employment, smoking, physical activity, alcohol intake, BMI, QOL, etc.	Bother from hot flushes was significantly decreased by the treatment.
Cardiovascular	Blood pressure	Campbell et al. [162]	A waitlist-controlled longitudinal study	Female post-treatment cancer patients (*n* = 70, 100% female, mean age, 53)	MBSR for eight weeks	Waitlist	Age, SBP, DBP	In the MBSR group, women with ‘higher BP’ at week 1 had decreased their SBP by week 8. In the MBSR group, decreases in rumination correlated with decreases in SBP and increases in mindful attention.
ANS, cardiovascular	Blood pressure, HRV	Muthukrishnan et al. [163]	RCT	Pregnant Indian women at 12 weeks gestation (*n* = 74, 100% female, mean age, 22)	Mindfulness meditation for five weeks	Usual obstetric care for five weeks	SBP, DBP, RR, perceived stress, HRV, cold pressor SBP, cold pressor DBP, etc.	In the meditation group, a significant decrease in perceived stress scores, a significant decrease in blood pressure response to the cold pressor test and a significant increase in HRV.
Cardiovascular	Blood pressure	Rakshani et al. [164]	RCT	Pregnant women at 12 weeks gestation with previous medical history in pregnancy (*n* = 68, 100% female, mean age, 27)	Yoga (breathing, meditation, yogi postures) for 15 weeks	Standard care plus conventional antenatal exercises (walking) for 15 weeks	Age, education, income, weight, height, BMI, SBP, DBP	A significant difference between groups in the ratio of pregnancy-induced hypertension.
Cardiovascular	Blood pressure	Thornton et al. [165]	RCT	Healthy community-dwelling women (*n* = 34, 100% female, mean age, 48)	Tai chi for 12 weeks	Control	Age, body weight, body height, blood pressure	Both systolic and diastolic blood pressure were significantly decreased by tai chi training.
ANS	HRV	Trivedi et al. [166]	RCT	Healthy women (*n* = 36, 100% female, mean age, 33)	Active meditation (breathing, positive emotions, guided imagery) for 20 min	Control (silence meditation—breathing only) for 20 min	Age, HRV, affect	In the experimental group, HRV (specifically, PNS) parameters showed a significant improvement compared to the control group.
ANS	HRV	Praveena et al. [167]	A prospective longitudinal study	Women within five years of menopause (*n* = 67, 100% female, age range, 45~60)	Yoga for three months	Control	Age, duration of menopause, body fat, resting heart rate, systolic blood pressure, etc.	Yoga practice improved HRV in early postmenopausal women significantly.
ANS	HRV	Audette et al. [168]	RCT	Sedentary women (*n* = 27, 100% female, mean age, 71)	Tai chi for 12 weeks (RCT)	Brisk walking for 12 weeks (RCT), sedentary life style for 12 weeks (a separate group)	Age, weight, exercise test, HRV, flexibility, single leg balance	In the tai chi group, significant improvement in estimated VO_2_ max, increase in high-frequency power (representing increased parasympathetic activity) and decrease in low-frequency power (representing decreased sympathetic activity) were found.
HPA	Cortisol	Field et al. [169]	RCT	Prenatally depressed women at 22 weeks gestation (*n* = 92, 100% female, mean age, 24)	Yoga for 12 weeks	Social support for 12 weeks	Age, education, SES, ethnicity, marital status	Cortisol levels decreased for both groups following each session.
HPA	Cortisol slope, stress, QOL	Carlson et al. [170]	RCT	Distressed survivor women of stage I to III breast cancer (*n* = 271, 100% female, mean age, 55)	MBCR for eight weeks	SET for 12 weeks, control (one-day stress management)	Age, cancer severity, time since diagnosis, alcohol, nicotine intake, quality of sleep, diet	Cortisol slopes were maintained over time in both the SET and MBCR groups relative to the control group, where the cortisol slopes became flatter. The MBCR group exhibited a significant improvement in stress symptoms and QOL compared to the SET group and the control group.
HPA	Cortisol	Daubenmier et al. [171]	RCT	Overweight/obese women (*n* = 47, 100% female, mean age, 41)	A four-month mindfulness program for stress eating	Waitlist	Age, weight, BMI, waist circumference, psychological state, CAR response, eating behavior	The mindfulness group exhibited significant reductions in the CAR and maintained body weight, while the control group had a stable CAR and gained weight. Improvements in mindfulness, chronic stress and CAR were associated with reductions in abdominal fat.
HPA, immune	Cortisol, cytokine	Witek–Janusek et al. [172]	A longitudinal study	Women newly diagnosed with early-stage breast cancer (*n* = 66, 100% female, mean age, 55), women without cancer (mean age = 55, *n* = 30)	MBSR for eight weeks	Non-MBSR, cancer-free group	Age, assessment time of the day	Women in the MBSR group had reduced cortisol levels, improved QOL and increased coping effectiveness compared to the non-MBSR group. The non-MBSR group exhibited continued reductions in NKCA and IFN-γ production with increased IL-4, IL-6 and IL-10 production, while the MBSR group re-established their NKCA and cytokine production levels.
Immune	Cytokine	Robins et al. [173]	RCT	Women with high CVD risk (*n* = 63, 100% female, 35–50 years)	Tai chi for eight weeks	Waitlist	Age, waist circumference	The tai chi group significantly lowered the level of interferon gamma, TNF, IL-8 and IL-4 compared to the control group.
Immune	Cytokine	Harkess et al. [174]	RCT	A subsample (*n* = 28, mean age, 41) from a population of women reporting psychological distress (*n* = 116, 100% female)	Yoga for eight weeks	Waitlist	Age, weight-to-height ratio	Reduced methylation of the TNF region in the yoga group relative to the waitlist control.
Immune	Cytokine	Gallegos et al. [175]	A longitudinal study	Trauma-exposed women (*n* = 50, 100% female, mean age, 44)	MBSR for eight weeks	N/A	Age, race, employment status, income	Session attendance was associated with significant decreases in IL-6 levels.

Abbreviation: RCT, randomized controlled trial; N/A, not available; PTSD, post-traumatic stress disorder; MBSR, mindfulness-based stress reduction; MBCT, mindfulness-based cognitive therapy; BMI, body mass index; QOL, quality of life; BP, blood pressure; SBP, systolic blood pressure; DBP, diastolic blood pressure; RR, respiratory rate; HRV, heart rate variability; ANS, autonomic nervous system; PNS, parasympathetic nervous system; MBCR, mindfulness-based cancer recovery; SET, supportive–expressive group therapy; HPA, hypothalamus–pituitary–adrenal; CAR, cortisol awakening response; IL, interleukin; TNF, tumor necrosis factor.

### 3.2. Changes in Mental Health Caused by Mind–Body Intervention and Findings in Women

The application of various mind–body interventions has shown improvement in mental health. In 23 randomized controlled trials (RCT) with 1373 participants, mind–body intervention (meditation, yoga, mindfulness) reduced depression, anxiety and stress with a moderate effect size [176]. When analyzed with RCTs using only active controls, the effect size was reduced [176]. To investigate whether physical yoga exercise alleviates depressive symptoms in people diagnosed with mental disorders, 13 RCTs investigating the effects of yoga intervention on the symptoms of 632 adults (female, 67.7%; mean age, 37.4 years) with a recognized diagnosed mental disorder according to DSM-3, -4, -5 were meta-analyzed. The yoga group had significantly reduced depressive symptoms compared to the control groups. Higher frequency of yoga sessions was associated with a greater reduction in depression [177]. In a meta-analysis study of 47 RCTs (3515 participants) using meditation and active controls, meditation reduced anxiety and depression with moderate effect size compared to active controls [143]. In a meta-analysis of seven studies to investigate the effects of qigong on depressive symptoms, qigong was effective in improving depression [178].

This application of mind–body intervention improves mental health in various populations. In a meta-analysis of 19 studies with 1076 participants (mean age, 71.8 years; female, 67.2%) to investigate the influence of mindfulness meditation (MM) intervention on depression in older adults, MM (structured MM, mean 7 weeks, 1.3 sessions/week, 102 min/session; unstructured MM, 4.7 sessions/week, 24.6 min/session) significantly improved depression compared to the control [179]. MM with guided meditation further reduces depression more than MM alone [179]. To investigate the effectiveness of meditative movements (tai chi, qigong, yoga) on major depressive disorder treatment, a meta-analysis of 15 RCTs (participants: MDD, 94.5%, female, 74.1%, mean age, 44.1 years) was performed and the results showed a significant improvement in depression (15 RCTs, *n* = 830) and anxiety severity (5 RCTs, *n* = 356) [180]. To investigate the effects of mind–body intervention on students who may be stressed due to academic demands, a meta-analysis of 34 studies (3296 students) using meditation and cognitive behavioral programs was performed to survey stress, depression and anxiety [181]. In a meta-analysis, mind–body intervention significantly reduced the stress, depression and anxiety levels of the students [181].

In particular, research on women suggests that mind–body intervention helps improve mental health of the female population of various ages and backgrounds. In a meta-analysis of six RCTs involving 375 pregnant women aged 20 to 40 to determine validity of yoga interventions in the management of antenatal depression, yoga interventions significantly reduced depression levels compared to the control groups (standard prenatal care, standard antenatal exercises, social support, etc.) [152]. Furthermore, they investigated two different variations of yoga training: physical exercise-based yoga and integrated yoga (addition of meditation or deep relaxation elements to physical exercises). Interestingly, significant reductions in depression levels were observed only in the integrated yoga group [152]. In a meta-analysis to investigate the effects of yoga on menopausal symptoms of women, 13 RCTs with 1306 menopausal women were included [182]. Regarding psychological factors (anxiety, depression), yoga intervention showed reductions in those symptoms compared to the control group, but the effects were comparable when compared with the exercise group. In another meta-analysis of 16 RCTs with 930 breast cancer patients for the investigation of effects of yoga, yoga intervention significantly reduced depression and anxiety compared to the control [183]. In the subgroup analysis, the reduction of anxiety was only significant when the practitioners continued practicing for more than three months. Mindfulness-based meditation has also been researched for its effects on breast cancer patients. In a meta-analysis which investigated the effects of mindfulness-based stress reduction (MBSR) and mindfulness-based cognitive therapy (MBCT) on 1709 breast cancer patients in ten studies, the intervention significantly reduced the levels of stress, anxiety and depression [154]. After completion of the intervention, the improved state of mind was maintained for a further six months for anxiety and 12 months for depression.

Other studies examining gender differences on the effects of mind–body intervention on mental health suggest that in various groups, such as adolescents, adults and drug addicts, women gain more benefits than men from the intervention. In an RCT which investigated whether school-based mindfulness training affects affective outcome differently depending on gender among early adolescents (sixth-grade students), participants who practiced mindfulness meditation 4–5 times weekly for six weeks (*n* = 52, mean age, 11.73 years, 44% female) and people in the active control group (*n* = 48, mean age, 11.85 years, 48% female) were compared and the analysis was performed according to gender. Female meditators exhibited a greater increase in positive feelings compared to the controls while male meditators exhibited equivalent gains with the control males. Furthermore, this improvement in feelings in females was associated with self-reported self-compassion, but not in males [155]. In order to see if meditation training has different effects on negative feelings depending on gender, 77 college students (46% female, mean age, 20.7) conducted a 12-week meditation training, which showed a greater decrease in negative feelings and a greater increase on the mindfulness and self-compassion scales in women compared to those in men [156]. This suggests that mind–body intervention may have a greater effect on women. In a study to find out the possibility and efficacy of using integrative qigong meditation as a treatment for substance abuse, 284 participants in the adult rehabilitation unit of a residential addiction treatment facility underwent a four-week qigong program. The results indicated that female meditation participants exhibited more significant reductions in anxiety and withdrawal symptoms than other groups [157].

### 3.3. Changes in Brain Structures and Functions by Mind–Body Intervention

The fact that mental and physical intervention structurally and functionally changes the brain is clearly demonstrated by accumulated brain imaging studies. In a meta-analysis study on the effects of meditation on brain structure [184], 300 meditation participants and 123 brain morphology differences were analyzed through 21 neuroimaging studies. In meditators’ brains, changes were consistently observed in the following brain function–related regions including eight brain regions: meta-awareness (frontopolar cortex/BA10), exteroceptive and interoceptive body awareness (sensory cortices; insula), memory consolidation and reconsolidation (hippocampus), self- and emotion regulation (anterior and middle cingulate; orbitofrontal cortex) and intra– and interhemispheric communication (superior longitudinal fasciculus; corpus callosum).

#### 3.3.1. Structural Changes in the Prefrontal Cortex by Mind–Body Intervention

The ventromedial prefrontal cortex (vmPFC) includes BA10, 14, 25, 32, 11, 12, and 13. In alert, non-stress conditions, the vmPFC controls subcortical structures (such as the amygdala, the nucleus accumbens, the hypothalamus), regulating emotional responses and habits [185,186,187]. However, in stress conditions, the control of the prefrontal cortex over the subcortical structure weakens, causing the amygdala to activate stress pathways in the hypothalamus and brain stem, resulting in excess secretion of noradrenaline and dopamine, which in turn weakens PFC control and strengthens amygdala activity, entering a vicious cycle [188]. Accumulated evidence has shown that the performance of mind–body interventions, including meditation, changes the structure and function of the frontal cortex. This supports improvements in stress management by mind–body intervention at the structural/functional level of the brain.

A meta-analysis by Fox et al. [184] showed changes in brain structures, especially increases of the gray and white matter in the prefrontal cortex, by meditation. Specifically, in the anterior prefrontal cortex (BA10), cortical thickness significantly increased compared to controls [189,190,191]. In the orbitofrontal cortex (BA11), cortical thickness [190,191] and white matter fiber density [190] were significantly increased in meditators compared to those in control groups. In the dorsolateral prefrontal cortex (BA46), white matter fiber density was significantly increased in meditators compared to controls [190]. Increase of cortical thickness was also observed in other regions of the frontal brain [192,193].

#### 3.3.2. Functional Changes in the Prefrontal Cortex by Mind–Body Intervention

A meta-analysis using 78 functional neuroimaging (fMRI, PET) studies from 31 meditation experiments involving 527 participants [194] investigated the effects of meditation on brain function. In this analysis, the following meditation types were included: focused attention (FA) (seven experiments [195,196,197,198,199,200,201]), mantra recitation (eight experiments [201,202,203,204,205,206,207,208]), open monitoring (OM) (ten experiments [196,197,209,210,211,212,213,214,215]), compassion/loving–kindness (six experiments [197,199,209,216,217,218]). Meta-analysis results showed that the frontal brain regions were activated in most of the meditation types. In the meta-analysis of FA meditation studies, the meditation group significantly activated the prefrontal cortex area including the premotor cortex (BA6), dorsal anterior cingulate cortex (BA24), dorsolateral prefrontal cortex (BA8/9) and left mid insula (BA13) [194]. In the meta-analysis of mantra recitation meditation studies, the meditation group exhibited significant activity in frontal regions including the posterior dorsolateral prefrontal cortex/left premotor cortex (BA6/8), presupplementary motor cortex and supplementary motor cortex (BA6) compared to the control group. In the meta-analysis of OM meditation studies, the meditation group significantly activated frontal regions including the left inferior frontal gyrus (BA44/45), presupplementary motor area (BA32/6), supplementary motor area (BA6) and motor cortex (BA6) compared to those of the control group [194]. In the meta-analysis of loving–kindness and compassion meditation studies, meditation induced significant activation in the right anterior insula/frontal operculum (BA13) which are part of the vmPFC. This region is related to awareness of bodily sensations and feelings [194].

#### 3.3.3. Changes in Functional Connectivity between the Prefrontal Cortex and the Amygdala by Mind–Body Intervention

During emotional regulation, the medial prefrontal regions directly influence the amygdala [219], while the lateral prefrontal cortices indirectly regulate the amygdala via the medial prefrontal and orbitofrontal system [220,221]. At rest, high anxiety negatively correlates with functional connectivity between the amygdala and the ventral medial PFC (mPFC), while low anxiety positively correlates with it [222]. Mind–body intervention affects emotional control by changing the functional connectivity between these medial prefrontal regions and the amygdala. According to a meta-analysis of 21 fMRI studies on eight-week MBSR and MBCT programs, these mind–body interventions reduced the functional activity of the amygdala, increased the functional connectivity between the amygdala and the prefrontal cortex and facilitated deactivation after the exposure to emotional stimuli [223], supporting the improvement of emotional regulation by mind–body intervention. In another RCT, meditation-naïve healthy adults (*n* = 67) were randomized into eight–week MBSR or a health enhancement program group. Affective pictures were shown to participants. The results showed a significant improvement in the functional connectivity between the amygdala and the vmPFC (emotion regulation-related regions) from exposure to affective pictures in the MBSR group compared to the other group. Moreover, meditation training was related with lower amygdala reactivity to positive pictures [224]. In another study, fMRI analysis was used to compare the response of participants conditioned with two weeks of mindfulness-based attention-to-breath meditation versus passive viewing in untrained participants. Meditation training was found to reduce amygdala activity compared to passive viewing while increasing emotion-related functional connectivity of the amygdala for the dorsal left prefrontal cortex [225]. In another study, Sant Mat meditators (*n* = 21, 67% female) exhibited a stronger positive functional connectivity between the amygdala and the ventrolateral prefrontal cortex to explicit happiness compared to the control group (*n* = 20, 60% female) [226]. These examples suggest that amygdala–prefrontal cortex integration is a potential neural pathway of emotional regulation by mind–body intervention. 

#### 3.3.4. Gender Differences in Brain Structural Changes by Mind–Body Intervention

To determine whether the meditation-induced hippocampus-specific effects are gender-dependent, analysis of high-resolution magnetic response data of 30 long-term meditation performers (50% female) and 30 well-matched control subjects (50% female) was conducted. The effect of meditation between men and women was found to be different in magnitude, laterality and location on the hippocampal surface [158]. Longitudinal studies are needed to determine whether this is a genetic difference or one induced by mediation in the male and female brains.

### 3.4. Changes in Cortisol Secretion by Mind–Body Intervention

With regard to the effects of mind–body interventions on human endocrine systems, the HPA axis has been the most widely explored [150]. In various populations such as colon cancer patients [227], breast cancer patients [172], prostate cancer patients [228], depressive patients [229] and healthy people [230,231], mind–body intervention affects regulation of the HPA axis which reflects stress levels. According to a meta-analysis of RCT studies investigating the effectiveness of meditation on physiological stress markers compared to active controls (seven studies, *n* = 212), the meditation group had significantly reduced cortisol levels compared to the control group [232]. According to a meta-analysis study investigating whether the effects of meditation on cortisol secretion levels vary depending on the level of stress [233], meditation intervention showed a significant blood cortisol reduction compared to control groups in ten independent studies (*n* = 336). Interestingly, this effect was only present in at-risk samples (patients with somatic illness). Twenty-one studies using saliva samples (*n* = 1063) showed significant cortisol reduction only in groups living in stressful life situations [233]. These patterns suggest benefits of meditation intervention, especially in at-risk populations.

In a meta-analysis of the effects of mindfulness mediation on the circadian rhythm of cortisol secretion (nine studies, *n* = 699) [234], there were no changes in cortisol awakening response (two studies [235,236]), 0 min post-awakening cortisol (one study [236]), 30 min post-awakening cortisol (two studies [236,237]), mean diurnal cortisol (one study [238]), pre-bedtime cortisol (two studies [236,237]) and pre-lunch cortisol (one study [237]), while there were significant reductions in diurnal cortisol—high slope (two studies [235,236]) and diurnal cortisol—low slope (three studies [235,236,237]) by meditation. According to a meta-analysis on the effects of yoga and MBSR on cortisol secretion (eight studies, *n* = 614) [239], yoga intervention significantly reduced waking cortisol (five studies [170,240,241,242,243]), afternoon salivary cortisol (three studies [170,240,244]), evening cortisol (five studies [170,240,241,242,243]), while it had no effect on salivary cortisol at 30 min post-awakening (three studies [240,241,242]) and 60 min post-awakening (two studies [241,242]), mid-morning cortisol (three studies [169,243,245]) and cortisol slope (three studies [170,240,241]). Cortisol secretion, the end product of the HPA axis activated by stress, affects vascular health and the immune system, and its chronic secretion by prolonged activation of the HPA axis also affects brain structure and function, affecting mental health.

Mind–body intervention also seems to significantly change the secretion of cortisol by contributing to the regulation of the HPA axis in studies conducted only on women. In an RCT for overweight/obese women (*n* = 47), a four-month mindfulness program for stress eating significantly reduced the cortisol awakening response compared to the waitlist [171]. In a 12-week RCT that examined the effects of yoga and social support on 92 prenatally depressed women, both groups had significantly reduced cortisol levels per session [169]. In an RCT where 271 distressed breast cancer survivors were randomized into a mindfulness-based cancer recovery (MBCR) group, a supportive–expressive group therapy (SET) group or a control group (stress management seminar), the MBCR and SET groups exhibited a more normative diurnal cortisol profile, whereas the control group exhibited a flat cortisol slope [170]. 

### 3.5. Changes in Blood Pressure, Heart Rate and Heart Rate Variability by Mind–Body Intervention

The stress response can initially activate both the sympathetic nervous system and the parasympathetic nervous system simultaneously, but then, as the parasympathetic nervous system is withdrawn, uninhibited sympathetic nerve activation maintains the heart rate increase [246]. Furthermore, mind–body intervention seems to increase overall heart rate variability (HRV) and decrease the blood pressure and heart rate by activating the parasympathetic nervous system [149,247] (Figure 1).

#### 3.5.1. Resting Systolic Blood Pressure

In a meta-analysis by Pascoe et al., meditation significantly reduced systolic blood pressure (SBP) (11 studies, *n* = 582) [232]. In a sub-analysis, automatic self-transcending (AST) meditation (three studies, *n* = 151 [248,249,250]) and FA meditation (three studies, *n* = 72 [251,252,253]) reduced resting SBP compared to the active control, while OM meditation did not reduce SBP (five studies, *n* = 359 [254,255,256,257,258]). In a meta-analysis of yoga interventions [239], yoga interventions significantly reduced resting SBP (17 studies, *n* = 1058 [242,245,252,257,259,260,261,262,263,264,265,266,267,268,269], while MBSR interventions did not change the resting SBP (two studies, *n* = 76) [257,262]. In a meta-analysis study of the effects of yoga on patients with coronary heart disease [270], yoga interventions of 3–6 months significantly reduced SBP compared to usual care (three studies, *n* = 330 [271,272,273]). In a meta–analysis study of the effectiveness of tai chi on risk factors in CVD for adults with essential hypertension [274], the tai chi intervention significantly decreased SBP (significant for all <three months, ≥three months, ≥six months) compared to the controls (15 studies, *n* = 772). In the meta–analysis of studies investigating the effectiveness of mindfulness training for adults with CVD [275], the mindfulness-based intervention groups had a significantly reduced SBP compared to the control groups (seven studies, *n* = 509 [258,276,277,278,279]), but no significant changes were observed in diastolic blood pressure (DBP) (six studies, *n* = 492 [258,276,277,278,279]).

#### 3.5.2. Resting Diastolic Blood Pressure

In a meta-analysis for resting DBP [232], it was significantly reduced when all meditation types were analyzed (11 studies, *n* = 582) [248,249,250,251,252,254,255,256,257,258], but it was not significant when analyzed according to the meditation subtype (AST (three studies, *n* = 151) [248,249,250], FA (three studies, *n* = 72) [251,252,253], OM (five studies, *n* = 359) [254,255,256,257,258] meditations). In a meta-analysis of yoga and MBSR (16 studies, *n* = 887), yoga intervention significantly reduced resting DBP compared to the active control, and there was no difference in effectiveness depending on the type of yoga. When comparing MBSR and non-MBSR (yoga) groups, both had a significantly reduced resting DBP [239]. In a meta-analysis study of the effects of yoga on patients with coronary heart disease [270], yoga interventions for three to six months significantly reduced DBP compared to usual care (three studies, *n* = 330 [271,272,273]). In a meta-analysis study investigating the effectiveness of tai chi on risk factors of CVDs [274], tai chi intervention did not change DBP in the interventions of less than three months (three studies, *n* = 194), but changed DBP in the interventions of more than three months (six studies, *n* = 620) or six months (six studies, *n* = 729). In a meta-analysis to investigate the effectiveness of qigong [178], diastolic blood pressure was found to be significantly decreased.

#### 3.5.3. Ambulatory Systolic Blood Pressure

In a meta-analysis for ambulatory SBP [232], it was significantly decreased in the total meditation analysis (five studies, *n* = 377) [254,280,281,282]. In an analysis depending on the meditation type, OM meditation significantly decreased the ambulatory SBP (three studies, *n* = 226) [254,281,282], while AST did not result in significant changes (two studies, *n* = 126) [283,284,285]. In a meta–analysis of the effects of yoga interventions on ambulatory SBP, yoga interventions did not significantly change the ambulatory SBP (three studies, *n* = 272) [283,284,285].

#### 3.5.4. Ambulatory Diastolic Blood Pressure

In a meta-analysis for ambulatory DBP [232], it was significantly reduced in the total meditation analysis (five studies, *n* = 352) [254,280,281,282,286], but there was no significant change in the analysis according to the type of meditation (AST (two studies, *n* = 126) [280,286], OM (three studies, *n* = 226) [254,281,282]). In a meta-analysis of the effects of yoga on ambulatory DBP (24 hours) [239], there was no change by yoga interventions (three studies, *n* = 242 [283,284,285]).

#### 3.5.5. Heart Rate

In a meta-analysis for HR (nine studies, *n* = 345) [232], OM meditation reduced the resting HR (five studies, *n* = 24), while FA meditation did not affect it. When all meditation groups were analyzed together, the resting HR decreased significantly. However, ambulatory HR was not changed by the intervention. In a yoga meta-analysis study (15 studies, *n* = 879) [239], yoga intervention significantly reduced the resting heart rate compared to the active control.

#### 3.5.6. Heart Rate Variability, Respiration Rate, Arterial Pressure

In a meta-analysis for the effects of yoga (four studies, *n* = 367) [239], both low and high frequencies of resting HRV were significantly changed by the yoga intervention compared to the controls. In the study to investigate whether long-term meditators exhibit difference in resting respiration rate compared to meditation-naïve people (*n* = 69), long-term mindfulness training exhibited a slower baseline respiration rate (RR) compared to the matched group of non-meditators. Regardless of age and gender, longer practice experience was associated with slower RR. This association was specific in intensive retreat practice but not in routine daily practice [287]. In a meta-analysis of yoga studies [239], yoga interventions (Iyengar yoga, hatha yoga/meditation, integrated yoga, yoga/lifestyle modification) significantly reduced the resting mean arterial pressure (five studies, *n* = 315). Mind–body intervention studies conducted only on women also showed significantly changed HRV. In a study comparing HRV and mood of women in an active meditation group with those in a breath-focused silence group, active meditation significantly increased HRV parameters (*n* = 36) and positive feelings (*n* = 48) compared to the control group [166].

### 3.6. The Effects of Mind–Body Intervention on Lipid Profile and Blood Glucose

The following studies suggested that mind–body interventions such as tai chi, yoga and meditation have positive effects on the lipid profile, blood glucose and insulin resistance, which is estimated to have a positive effect on cardiovascular health. In a meta-analysis to examine the effect of tai chi on cardiovascular disease risk factors for adults with essential hypertension, tai chi practice significantly reduced total cholesterol, triglycerides, LDL-C (five studies, *n* = 846), as well as blood glucose (four studies, *n* = 612) [274]. In a meta-analysis that examined the effect of yoga on the lipid profile (four studies, *n* = 332), yoga practice significantly reduced TG and increased HDL-C and did not change the levels of LDL-C and TC as compared to the usual care [270]. In another meta-analysis which examined the effects of meditation on physiological markers of stress (four studies, *n* = 328), meditation significantly reduced TG but did not change the levels of HDL, LDL and cholesterol as compared to active controls [232]. 

Several studies also focused specifically on investigating the effects of mind–body interventions on blood lipids and glucose in women. In an RCT which examined the effects of yoga therapy on glucose metabolism and blood lipids of 90 adolescent girls with polycystic ovary syndrome (age = 15–18 years), both yoga therapy and conventional physical exercise significantly reduced fasting blood glucose, LDL, TC, TG and increased HDL, with more significant improvements in yoga compared to conventional physical exercise [288]. In another RCT on 37 prediabetic females (diabetes risk score ≥ 60, mean age, 52), the yoga group exhibited a significant decrease in plasma glucose levels after three months of yoga compared to the non-practicing group [289].

### 3.7. Changes in Inflammatory Response Levels by Mind–Body Intervention

Recent findings include inflammation to a set of existing well-known cardiovascular disease risk factors such as high blood pressure, high cholesterol and diabetes [290,291]. The following studies suggested that mind–body interventions may partially contribute to the prevention of cardiovascular disease also by pathways affecting the immune system in addition to the other risk factors of cardiovascular disease discussed above. According to a systematic review of 20 randomized controlled trials researching mindfulness meditation (comprising more than 1600 participants), it is provisional for mindfulness meditation to be related with inflammation (reductions in NF-kB transcription activity and CRP level); however, the association has been shown to be replicated [292]. While acute psychological stress activates NF-kB in peripheral blood mononuclear cells [121,293,294,295,296,297], the use of mindfulness meditation reduced the expression of NF-kB in RCTs for breast cancer patients (*n* = 71) [295], lonely older adults (*n* = 40) [296] and older adults (*n* = 49) [297]. In RCTs in patients with ulcerative colitis (*n* = 55) [298] as well as in healthy people with inflammatory risk markers (*n* = 40 [296], *n* = 185 [238]), mindfulness meditation also induced a reduction [298] or a tendency of reduction [238,296] in the level of CRP, a representative inflammatory protein. In another meta-analysis, meditation reduced TNF-α compared to active controls (three studies, *n* = 100) [232], and IL-6 was significantly decreased by yoga or MBSR intervention when compared to active control groups (four studies, *n* = 128) [239]. However, a meta-analysis of MBSR only showed no significant IL-6 changes compared to the controls (five studies, *n* = 125) [232].

In studies of women only, mind–body intervention has significant effects of reducing inflammation. DNA methylation was investigated using peripheral blood samples of 116 women reporting psychological distress in an RCT setting. Eight weeks of yoga intervention showed a significant difference in methylation in the TNF region as a whole as compared to the waitlist control group, and no significant differences were found in other genes. This suggests the effects of yoga interventions on the immune system at the epigenetic level [174]. In addition, in a pilot study of the effect of the MBSR program on inflammatory biomarkers in women with interpersonal trauma, eight-week MBSR significantly reduced IL-6 levels (*n* = 50) [175].

### 3.8. The Effects of Mind–Body Intervention on Menopausal Symptoms Related with Cardiovascular Health

In women, the menopause transition period is associated with an increase in CVD risk factors as described in Section 1.4. It has been known that mind–body interventions provide relief towards women’s overall menopausal symptoms. In a meta-analysis of 13 RCTs involving 1306 women [182], yoga reduced total menopausal, psychological, somatic, vasomotor and urogenital symptoms compared to the non-treatment group. Compared to exercise controls, significant changes were observed only for vasomotor symptoms. In an RCT which compared the effects of MBSR (*n* = 98) or active controls (menopause education control, MEC, *n* = 99) on menopause-related symptoms for perimenopausal and postmenopausal women at the baseline and at various time periods post-intervention [159], both groups exhibited a reduced total Greene Climacteric Scale (GCS) score at eight months. Compared with active controls, MBSR significantly reduced overall menopausal symptoms as well as two sub-scales of menopausal symptoms, i.e., anxiety and depression, but not other symptoms, i.e., somatic, urogenital and vasomotor symptoms. In another RCT in late perimenopausal and early postmenopausal women (*n* =110), bother and distress from hot flushes and night sweats were significantly reduced after three-month MBSR compared to the waitlist control group [161]. Symptoms of menopause are reduced by mind–body interventions, suggesting their contribution to lowering the menopause-associated cardiovascular disease risk in women.

## 4. Conclusions

There is a gender difference in the development, prognosis and mortality of CVD, and the underlying mechanism may include a female-specific vulnerability in stress–amygdala–physiological response, as studies have reported the association between amygdala activity and CVD-specific physiological response only in women [72,73,74,75]. Mind–body intervention improves stress management skills, which lowers the level of stress that the brain perceives. This characteristic of mind–body intervention is shown to reduce the risk of CVD by reducing the downstream mechanisms induced by stress, including activation in the amygdala, the HPA axis and the sympathetic nervous system. Since mind–body interventions also affect other aspects of cardiovascular disease such as hypertension, diabetes and dyslipidemia, synergistic effects of mind–body intervention on cardiovascular health are expected along with improved mental health [299]. Although there is not yet enough evidence of the specific effect of mind–body interventions on women, they are expected to be used as a preventive strategy to reduce the risk of women’s specific vulnerability in CVD, as the evidence has shown that it helps to reduce stress and improve stress-related cardiovascular physiology. In the future, a robust study design is required to demonstrate the effects of mind–body intervention customized for women in the prevention or treatment of CVDs.
